# Poly(lactide)-g-poly(butylene succinate-co-adipate) with High Crystallization Capacity and Migration Resistance

**DOI:** 10.3390/ma9050313

**Published:** 2016-04-27

**Authors:** Xi Yang, Huan Xu, Karin Odelius, Minna Hakkarainen

**Affiliations:** Department of Fibre and Polymer Technology, School of Chemical Science and Engineering, KTH Royal Institute of Technology, SE-100 44 Stockholm, Sweden; xiy@kth.se (X.Y.); hihuan@kth.se (H.X.); Hoem@kth.se (K.O.)

**Keywords:** polylactide, poly(butylene succinate-co-adipate), plasticizing, crystallization, oxygen permeability, migration resistance

## Abstract

Plasticized polylactide (PLA) with increased crystallization ability and prolonged life-span in practical applications due to the minimal plasticizer migration was prepared. Branched plasticized PLA was successfully obtained by coupling poly(butylene succinate-co-adipate) (PBSA) to crotonic acid (CA) functionalized PLA. The plasticization behavior of PBSA coupled PLA (PLA-CA-PBSA) and its counterpart PBSA blended PLA (PLA/PBSA) were fully elucidated. For both PLA-CA-PBSA and PLA/PBSA, a decrease of *T*_g_ to around room temperature and an increase in the elongation at break of PLA from 14% to 165% and 460%, respectively, were determined. The crystallinity was increased from 2.1% to 8.4% for PLA/PBSA and even more, to 10.6%, for PLA-CA-PBSA. Due to the inherent poor miscibility between the PBSA and PLA, phase separation occurred in the blend, while PLA-CA-PBSA showed no phase separation which, together with the higher crystallinity, led to better oxygen barrier properties compared to neat PLA and PLA/PBSA. A higher resistance to migration during hydrolytic degradation for the PLA-CA-PBSA compared to the PLA/PBSA indicated that the plasticization effect of PBSA in the coupled material would be retained for a longer time period.

## 1. Introduction

Crystallization kinetics studies of plasticized polylactide (PLA) show that adding a plasticizer improves the ductility and also accelerates the growth rate of spherulite [1,2]. As a good example, blending 10 wt % low molecular weight poly(ethylene glycol) (PEG) into PLA increased crystallinity of PLA from 14% to 49% [3]. The crystallinity, in essence, depends on two factors: the crystal growth rate and the nucleation efficiency. Inorganic nucleating agent such as CaCO_3_ and TiO_2_ [4], organic nucleating agents like carbon nanotubes [5] and graphene oxide [6,7], and bio-based nucleating agent such as PDLA [8] and *myo*-inositol [9] all increase the amount of nucleation and therefore the crystallinity of PLA. In long chain branched PLA, the branching points acted as nucleation points for PLA [10]. This effect is, however, counteracted by the entanglements which disturb the folding of the molecular chains. The importance of the actual branching sites in the crystal formation is obvious even for low molecular weight branches. Acrylated poly(ethylene glycol) (Acrylated PEG) with a *M*_n_ of about 480 g/mol was grafted onto PLA improving the crystallization ability of the grafted PLA. It was determined that the PLA-graft-poly(acrylated PEG) acted as a nucleating agent and that branched PLA has a higher free volume due to the higher number of chain ends which led to higher chain mobility [11]. This effect was shown to level off and no correlation of increased crystallinity with a higher degree of branching was found. Similarly, when acrylated PEG with a molecular weight of 550 g/mol was grafted to PLA with content from 10 wt % to 40 wt %, the elongation at break of all the grafted PLA were improved and the glass transition temperature gradually decreased with increased acrylated PEG [12]. It was also shown that when PLA was grafted with 10 wt % acrylated PEG, the crystallinity was increased from 0.9% to 32.4%. However, when more acrylated PEG was grafted, the crystallinity decreased. In addition to crystallization control, branching modification provides an efficient approach to increase polymer melt strength [13], improve the interfacial adhesion between PLA and additives [14], and tune degradability and degradation products [15]. Therefore, a good combination of plasticization and nucleation could significantly improve the nucleation and crystal growth of PLA [16,17], mitigate the brittleness and adjust the degradation products pattern of PLA [18]. We have developed a method to covalently bind oligomeric poly(3-hydroxybutyrate) (PHB) to PLA through reactive extrusion to achieve plasticized PLA [18]. An unsaturated end of the plasticizer was covalently attached to the main chain of PLA by virtue of free-radical-initiated grafting and it drastically improved the ductility and crystallization ability and lowered the glass transition temperature. Besides, the covalently attached PHB showed higher migration resistance as compared to blended PHB. The increased migration resistance of plasticizers from the bulk is an important characteristic to achieve, which enables a longer service-life time of the final product.

Good miscibility between the PLA matrix and the added modifier is a vital parameter, since it affects the crystallization behavior, barrier properties, mechanical performance and thermal properties. Besides, phase separation between the additive and PLA matrix could be induced by bad miscibility. Phase separation between PLA and PBS or PBSA was observed in many studies [19,20,21,22,23,24]. Triblock copolymer of PLA and copoly(ester-urethane) (80 mol % PBS and 20 mol % poly(butylene azelate)) were synthesized and showed improved flexibility and good miscibility between the two components [25]. However, when the amount of copoly(ester-urethane) was higher than 68%, two glass transition temperatures could be observed. PLA/PBSA blends mixed with organically modified montmorillonite clays showed reduced size of dispersed PBSA phase, but the mechanical properties were still poor [26]. Triphenyl phosphite (TPP) was added to enhance *in situ* compatibilization between PLA and PBSA [27]. Depending on the quantities of TPP, PLA/PBSA blends showed improved toughness; however, high concentration of TPP will accumulate in the blends and therefore affect its thermomechanical properties.

Based on the above studies, it can be concluded that branched PLA has greater nucleation efficiency and subsequently exhibits enhanced crystallization ability as compared to linear PLA [10,11,12]. Plasticizer migration resistance could also be improved when branched plasticizers were used [18,28,29,30]. It has been shown that blending PBSA into PLA could facilitate crystallization of PLA and mitigate the brittleness of PLA when the phase separation could be reduced by adding compatibilizer or annealing the blends [20,27]. PBSA is also one of the main candidates from the biodegrable aliphatic polyesters family that could be used in food packaging field [31]. Therefore, here we aim at a fully biobased and biodegradable plasticized PLA with a low plasticizer migration rate, an enhanced crystallization ability and an increased oxygen barrier property with no phase separation between PLA and the PBSA plasticizer. To achieve this, we combined the above approaches, functionalized PLA with carboxylic groups using crotonic acid, a product from feedstock recycling of PHB [32], and subsequently coupled a low molecular weight PBSA onto the carboxylic groups to prepare PLA with a branched structure through grafted PBSA plasticizer.

## 2. Materials and Methods

### 2.1. Materials

Poly(lactide), PLA, was supplied by NatureWorks (Minnetonka, MN, USA) PLA (5200D) with *M*_n_ = 160,000 g/mol, *Đ* = 1.7 and l-lactide/d-lactide: 96/4. Crotonic acid (98%), 4-dimethylaminopyridine (≥99%, ReagentPlus), *N*,*N*-dimethylformamide (≥99%, GC grade) and dichloromethane (≥99.9%, GC grade) were purchased from Sigma Aldrich (Stockholm, Sweden) and used as received. Benzoyl peroxide (75%, remainder water) and heptane (≥99%) were supplied by Acros Organics (Geel, Belgium), toluene (HPLC grade) and chloroform (HPLC grade) were purchased from Fisher Scientific (Gothenburg, Sweden) and water (LiChrosolv^®^, Stockholm, Sweden, for chromatography) and methanol (LiChrosolv^®^, for chromatography) were supplied by Merck Millipore (Stockholm, Sweden) and used as received. Poly(butylene succinate-co-adipate) with *M*_n_ = 1900 g/mol was synthesized by step-growth polymerization according standard procedures. The monomers, 1,4-butanediol and succinic acid/adipic acid 1:1, were added to the reaction vessel with the total molar ratio of acid to alcohol of 1 to 1.2 The reaction vessel was immersing into a thermostated oil bath at 90 °C under nitrogen and the reaction temperature was stepwise increased to 190 °C. Titanium isopropoxide (TIP) was used as catalyst, the reaction temperature was increased to 220 °C and the reaction was allowed to proceed under reduced pressure. The reaction mixture was thereafter cooled to room temperature, the product was dissolved in chloroform and precipitated in ice cold methanol. After decantation, the product was dried under vacuum to constant weight.

### 2.2. Crotonic Acid Functionalized PLA

Grafting of crotonic acid to PLA was achieved through a free radical reaction under a N_2_ atmosphere. PLA was first dissolved in toluene under rapid stirring at 100 °C until complete dissolution. Crotonic acid dissolved in toluene was then added to PLA solution followed by the addition of the initiator, benzoyl peroxide. The mass ratio of PLA to crotonic acid and benzoyl peroxide was 10/1/0.06 (PLA/CA = 1 mol/186 mol). The relatively high amount of CA was added to promote grating reaction. After 12 h of reaction, the solution was concentrated, chloroform was added and crotonic acid grafted PLA (PLA-CA) was precipitated in heptane, washed with water and dried in a vacuum oven at room temperature to constant weight.

### 2.3. PBSA Plasticizer Coupled PLA

To couple PBSA plasticizer to PLA-CA, *N*,*N*′-dicyclohexylcarbodiimide was used as a coupling reagent and 4-dimethylaminopyridine as a catalyst for a Steglich esterification. PLA-CA and PBSA were dissolved in dichloromethane with a mass ratio of PLA-CA to PBSA of 100/19 (PLA-CA/PBSA = 1 mol/15 mol). The esterification was performed for 3 days at room temperature under a N_2_ atmosphere. PBSA coupled PLA-CA (PLA-CA-PBSA) was obtained after precipitation in diethyl ether, washing with methanol and then water. After purification, PLA-CA-PBSA was dried in the vacuum oven at room temperature until constant weight.

### 2.4. Film Formation

Neat PLA, PLA blended with PBSA (PLA/PBSA), and PLA-CA-PBSA films were prepared by solution casting. 5% (*w*/*w*) solutions of PLA, PLA/PBSA and PLA-CA-PBSA were prepared by heating the mixtures to 40 °C under stirring for 2 h. All solutions were thereafter cooled to room temperature, poured into a petri dish and dried for several days at room temperature. The films were then dried in a vacuum oven at room temperature to a constant weight. Melt pressing was subsequently preformed on the dry films. 3 g of material was placed in a 10 cm × 10 cm metal frame and pressed for 3 min at 180 °C with a pressing force of 200 kN.

### 2.5. Hydrolytic Degradation

After melt pressing, about 15 mg rectangular films of PLA, PLA/PBSA and PLA-CA-PBSA were put into vials filled with 10 mL chromatography water and closed with butyl/polytetrafluoroethylene septa and aluminum lids. After hydrolytic degradation at 60 °C for 2 and 4 weeks, the solutions, after removing the remaining solid, were mixed with methanol (water:methanol = 1:3 in volume) for further analysis.

### 2.6. Characterization of PLA, PLA/PBSA and PLA-CA-PBSA

The chemical structure of PLA-CA-PBSA was determined using proton nuclear magnetic resonance, ^1^H NMR. The NMR spectra were obtained by a Bruker Avance DPX-400 NMR instrument (Bruker, Zurich, Switzerland) operating at 400 MHz. Analysis was performed at room temperature using chloroform-d (CDCl_3_) as solvent.

Number-average molecular weight (*M*_n_), weight average molecular weight (*M*_w_), z-average molecular weight (*M*_z_) and dispersity index (*Đ*) of neat PLA, PLA-CA, PLA/PBSA and PLA-CA-PBSA films were evaluated with a Verotech PL-GPC 50 Plus size exclusion chromatography (SEC) system (Varian, Palo Alto, CA, USA). This SEC is equipped with a PL-RI detector and two PLgel 5 μm MIXED-D (300 × 7.5 mm) columns from Varian. Chloroform was used as mobile phase with toluene as an internal standard and polystyrene standards (from 162 to 371,100 g/mol) were used for calibration.

The thermal properties of the compression molded films were evaluated using a Mettler Toledo differential scanning calorimetry (DSC), 820 Module. A temperature program was set in which the samples were first heated from 20 to 200 °C, then cooled to −50 °C followed by a second heating scan to 200 °C at a heating speed of 10 °C/min under a nitrogen atmosphere. The melting temperature (*T*_m_), cold crystallization temperature (*T*_cc_) and glass transition temperature (*T*_g_) were determined from first and second heating scans. *T*_m_ was noted as the maximum of the melting peak and *T*_g_ as the mid-inflection point of the glass transition. Crystallinity (*χ_c_*) was calculated using the cold crystallization and melting enthalpies from the first heating process by using the following equation:

(1) $$\chi_{c}=\frac{\Delta H_{m}-\Delta H_{\text{cc}}}{\Delta H_{m}^{0}\times w_{\text{PLA}}}\times 100\%$$

∆*H*_m_ is the enthalpy of melting, ∆*H*_cc_ is the enthalpy of could crystallization and $\Delta H_{m}^{0}$ (93.1 J/g) is the enthalpy of melting 100% crystalline PLA and *w*_PLA_ is the weight percentage of PLA.

The formation and growth of PLA crystals in the films was observed by polarized optical microscopy. Thin films of PLA, PLA/PBSA and PLA-CA-PBSA were melted by a Mettler FP82HT hot stage at 200 °C for 3 min. The samples were then cooled rapidly to different temperature (*i.e.*, 100 and 120 °C). At these temperatures the PLA, PLA/PBSA and PLA-CA-PBSA films were isothermally crystallized for 30 min at which time a polarized optical microscopy (POM) equipped with a Leica digital camera was applied.

To identify the structural features for the crystalline developed in the isothermal crystallization, two-dimensional wide-angle X-ray diffraction (2D-WAXD) measurements were carried out using a home-made laboratory instrument (Bruker NanoStar, CuKα-radiation) in the Crystallography Lab, Department of Molecular Biology and Biotechnology, University of Sheffield. The X-ray beam with a wavelength of 0.154 nm was focused to an area of 4 μm × 4 μm, and the distance from sample to detector was held at 350 mm for WAXD measurements. An X-ray CCD detector (Model Mar 345, a resolution of 2300 × 2300 pixels, Rayonix Co. Ltd., Evanston, IL, USA) was employed to collect the 2D-WAXD images.

Mechanical properties of all materials were determined using an INSTRON 5944 module equipped with pneumatic grips. The measurements were performed using a load cell with a maximum load of 500 N at a crosshead speed of 20 mm/min. Films were cut into strips with a thickness of 0.13 ± 0.02 mm, width of 5 mm and a gauge length of 20 mm was used. The samples were preconditioned prior to being tested following the standard method described in ASTM D618 (Standard practice for conditioning plastics for testing, 40 h at 50% ± 5% relative humidity and 23 ± 1 °C). At least 8 specimens from each material were tested.

The morphology of the fractured surfaces was investigated by an SE-4800 scanning electron microscope (SEM) (Hitachi, Tokyo, Japan) after the PLA films were fractured in liquid nitrogen and sputter-coated with a 3.5 nm-thick Pt layer.

Oxygen permeability was evaluated at 23 °C with a Mocon Oxtran 2/20 instrument (Modern Controls Inc., Minneapolis, MN, USA) with coulometric sensor under the ASTM D3985 guidance (Standard test method for oxygen gas transmission rate through plastic film and sheeting using a coulometric sensor) at 0% and 50% relative humidity (RH). Films were sealed between aluminum foils and the exposed area was 5 cm^2^. The films thickness was measured to 0.13 ± 0.02 mm with a Mitutoyo micrometer.

After hydrolysis, the solutions containing the migrated water-soluble compounds were analyzed by electrospray ionization-mass spectrometry, ESI-MS. A Finnigan LCQ ion trap mass spectrometer (Finnigan, San Jose, CA, USA) in positive mode was used for the analysis with an ion source operating at 4.5 kV. The capillary heater was set to 200 °C. The flow rate of the syringe pump was 5 μL/min. Nitrogen was used as nebulizing gas.

## 3. Results and Discussion

Crotonic acid was firstly grafted onto PLA and subsequently a low molecular weight bioplasticizer, PBSA, was coupled to the crotonic acid functionalized PLA to simultaneously promote the flexibility and barrier properties of PLA, while avoiding problems with early plasticizer migration.

### 3.1. Synthesis of PLA-CA-PBSA

**Scheme 1:**
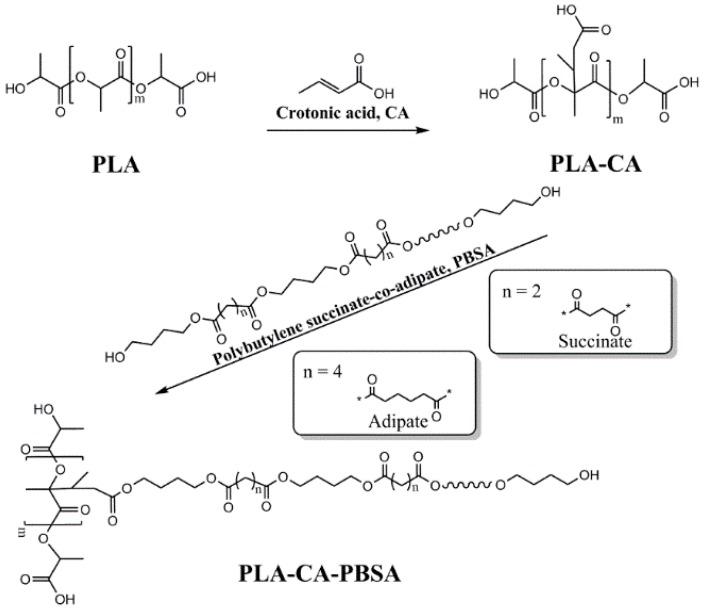
Schematic presentation of the most likely crotonic acid functionalization of PLA and PBSA coupling to PLA-CA.

**Figure 1:**
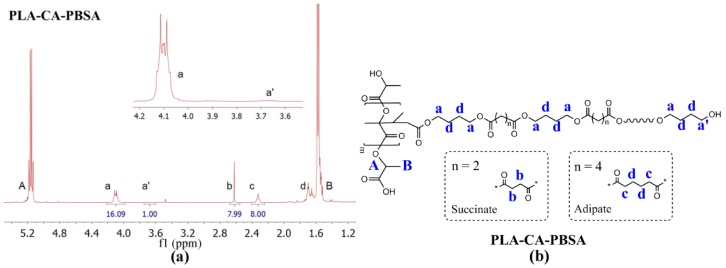
(**a**) ^1^H NMR spectrum of PLA-CA-PBSA; (**b**) The peak assignment for the structure.

Crotonic acid was firstly grafted to PLA through a free radical reaction, after which PBSA was coupled to the carboxylic-functionality of the CA grafted PLA, Scheme 1. The structural characteristics of PLA-CA-PBSA were determined by ^1^H NMR and as expected, the characteristic signals of both PLA and PBSA were observed in PLA-CA-PBSA, Figure 1. The synthesized PBSA has equal mole of succinic and adipic acid repeating units as determined by the intensity of proton *b* and *c* (2.6 and 2.3 ppm) corresponding to methylene proton next to carbonyl in succinic and adipic acid. The protons *a* (4.1 ppm) are assigned to the protons of butanediol units in the PBSA. At a shift of about 3.6 ppm, *a’*, signal of the protons next to hydroxyl end group in PBSA is seen. A successful esterification could be confirmed by the ratio between the integrals of peak *a* and *a’* which gives an indication of the ratio between repeating units and chain ends of PBSA. After esterification, PLA-CA-PBSA has a ratio of 16:1 of peak *a* and *a’* while PBSA before coupling has a ratio of 9:1.

The number average molecular weight (*M*_n_), weight average molecular weight (*M*_w_), z-average molecular weight (*M*_z_) and dispersity (*Đ*) of PLA, PLA-CA, PLA/PBSA and PLA-CA-PBSA were measured by SEC, Table 1. Minimal chain altering reactions occurred during the CA-functionalization and no unreacted crotonic acid was left after purification. This was confirmed by the fact that no obvious changes in *M*_n_ and *M*_w_ were seen for PLA-CA in comparison with PLA. PLA-CA-PBSA had the highest *M*_n_ and *M*_w_, indicating that PBSA was successfully coupled onto PLA-CA. This agrees with the conclusions drawn from ^1^H NMR. In addition, the almost unchanged *M*_z_ values confirm that no significant crosslinking took place, which was further supported by the full solubility of the grafted samples in chloroform. A low molecular weight fraction could, however, still be detected due to the existence of un-coupled PBSA. Less than 100% esterification efficiency was also shown by ^1^H NMR.

### 3.2. Characterization of Compression Molded Film PLA, PLA/PBSA and PLA-CA-PBSA

**Figure 2:**
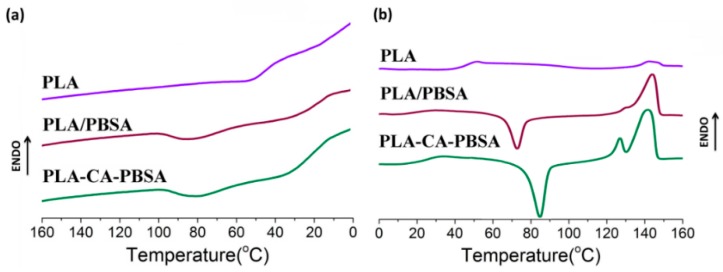
DSC trace of PLA, PLA/PBSA and PLA-CA-PBSA during (**a**) cooling and (**b**) second heating scan.

After compression molding, the thermal properties of PLA, PLA/PBSA and PLA-CA-PBSA were evaluated by DSC, Table 2 and Figure 2. During the second heating scan, neat PLA had a *T*_g_ at 44.3 °C, which decreased to 18.3 °C for the blend with 20 wt % PBSA. PLA-CA-PBSA, on the other hand, presented a *T*_g_ at 24.4 °C, which is slightly higher than the *T*_g_ of PLA/PBSA but still 20 °C lower than the *T*_g_ of neat PLA. This decrease indicates that introducing PBSA into PLA either through blending or coupling enhanced the chain mobility of PLA. In an earlier study, the *T*_g_ of PLA decreased with an increasing weight fraction of PBS (*M*_w_ = 1.2 × 10^5^ g/mol, *T*_g_ = −34 °C). Blending with 20 wt % PBS decreased the *T*_g_ of PLA by only 4 °C [33]. The larger reduction of *T*_g_ shown in the present study could be explained by the lower *T*_g_ (<−50 °C) of the synthesized PBSA plasticizer, which is caused by the combination of more flexible structure due to the adipic acid units and much lower molecular weight and agrees well with previous values reported for PLA plasticized with low molecular weight ester plasticizers [18,28]. Blending 20 wt % of glucose ester (*M*_n_ ≤ 1030 g/mol) with PLA could reduce *T*_g_ to about 25 °C, which was 30 °C lower than the *T*_g_ of neat PLA [28]. Blending PLA with 20 wt % oligomeric PHB led to a 29 °C decrease in *T*_g_ while grafting the same amount of oligomeric PHB resulted in a 25 °C decreasing compared to neat PLA [18].

Further, distinct melt and cold crystallization of PLA were observed in both PLA/PBSA and PLA-CA-PBSA. PLA/PBSA and PLA-CA-PBSA showed higher melting enthalpies for the PLA part as compared to the neat PLA during the second heating process. This indicates that PBSA improves the crystallization ability of PLA irrespective if the plasticizer is blended with or coupled to PLA. It was also illustrated previously that blending PBS could enhance the cold crystallization of PLA due to the enhanced molecular mobility [33,34]. Moreover, the molten droplets of polyesters with low molecular weight, as elucidated, could act as nucleation agents for PLA [22,35]. Similar crystallization enhancement phenomena, but with a different principle related to branched polymer, were reported previously [10,18,36,37]. The branching points on the PLA chain serve as nucleation points, leading to a higher ability for nucleation compared to neat PLA. PLA-CA-PBSA showed two melting peaks at 127.0 and 141.6 °C. The additional melting peak comparing to PLA and PLA/PBSA was presumably due to the generation of less-ordered crystals which involved imperfect chain packing of PBSA grafted PLA. Adding biodegradable PBSA into the PLA matrix is hence a means to tune the thermal properties and crystallization profiles while preserving the biodegradable nature of PLA.

**Figure 3:**
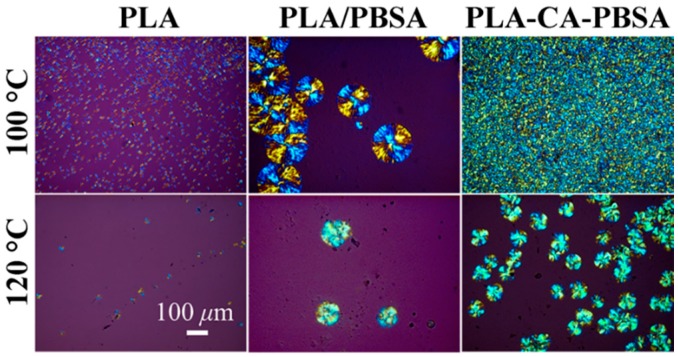
Crystalline morphology of isothermal crystallization at 100 and 120 °C of neat PLA, PLA/PBSA and PLA-CA-PBSA by polarized optical microscopy.

POM observations of the crystalline morphology developed in neat PLA, PLA/PBSA and PLA-CA-PBSA during isothermal crystallization at 100 and 120 °C, respectively, are presented in Figure 3. After isothermal crystallization for 30 min at 100 °C, the neat PLA showed a strong ability to nucleate, but the crystal growth rate was slow and therefore many and small spherulites were observed. PLA-CA-PBSA showed the strongest nucleation ability and after 30 min, spherulites covered the bulk. In clear contrast, the PLA/PBSA had weak nucleation ability but the spherulites grew quickly, leading to the formation of large and distinct spherulites. The influence and differences between blended PBSA and coupled PBSA are more clearly observed after isothermal crystallization at 120 °C. After 30 min of crystallization at 120 °C, PLA-CA-PBSA exhibited a strong nucleation ability, presumably due to the grafting points which acted as nucleation points [18,36,37] and big spherulites were observed, which was attributed to its improved chain mobility. Nucleation took place slowly in PLA/PBSA; however, once the crystallites started to grow, the growth rate was high. Compared to PLA, larger spherulites were observed in PLA/PBSA, but in smaller quantities. This is consistent with the low *T*_g_ in DSC measurement.

**Figure 4:**
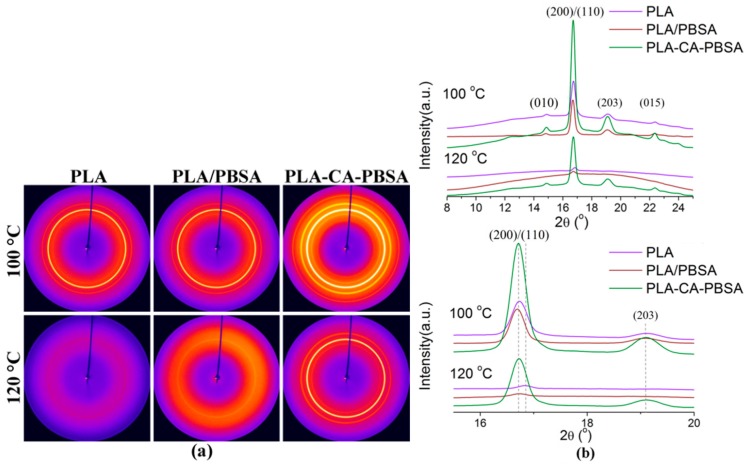
(**a**) 2D-WAXD patterns and (**b**) 1D-WAXD intensity profiles extracted from the corresponding 2D-WAXD patterns for PLA, PLA/PBSA and PLA-CA-PBSA.

The crystalline structure in PLA, PLA/PBSA and PLA-CA-PBSA after isothermal crystallization at 100 and 120 °C was identified by utilizing 2D-WAXD, Figure 4. Blending PBSA into PLA decreased the *T*_g_ which should significantly improve the chain mobility and probably facilitated the higher crystal growth rate observed by POM. However, due to the poor nucleation ability of PLA/PBSA, only weak diffraction rings were observed at 100 °C. An amorphous halo was observed in PLA/PBSA after crystallization at 120 °C. On the other hand, PLA-CA-PBSA exhibited strong diffraction rings which are attributed to the lattice planes (200)/(110), regardless of the isothermal crystallization temperature. The diffraction signals assigned to lattice planes (010), (203) and (015) were observed in all PLA-CA-PBSA samples. This supports that coupled PBSA accelerated the crystallization ability of PLA. 1D-WAXD intensity profiles were extracted from the 2D-WAXD. Typical peaks of PLLA α-crystal assigned to lattice planes (010), (200)/(110), (203) and (015) were located at 2θ = 14.8°, 16.7°, 19.0° and 22.3°, respectively [18,38]. In earlier studies, three strong diffraction peaks at 2θ = 19.3°, 21.5°, and 22.4° corresponding to (020), (021), and (110) planes respectively have been assigned for crystalline neat PBSA [39]. This provides evidence that the observed crystals originated from crystallization of PLA chains and PBSA was located in the amorphous regions.

The *T*_g_ of PLA is 20 °C higher than *T*_g_ of PLA-CA-PBSA, so to make a comparison at the same *T*_c_-*T*_g_, the crystallization of PLA at 120 °C can be compared to the crystallization of PLA-CA-PBSA at 100 °C. This comparison shows that 2θ reflection peaks of PLA-CA-PBSA had much higher intensity than those of PLA showing that PLA-CA-PBSA had much higher crystallinity than PLA even when both of them were crystallized at *T*_c_ 76 °C higher than their *T*_g_. It has been shown when PLA crystallizes at *T*_c_ < 120 °C, less ordered α’ crystals are formed. Compared to α-crystal, α’ lattice planes (200)/(110) and (203) have lower 2θ reflections [40] and this phenomenon was also observed here for the neat PLA samples. Crystals formed at 120 °C have higher 2θ reflection peaks than crystals formed at 100 °C. After PBSA was added into PLA matrix by either blending or grafting, 1D-WAXD indicates that less ordered crystals were formed after isothermal crystallization at both 100 and 120 °C.

**Figure 5:**
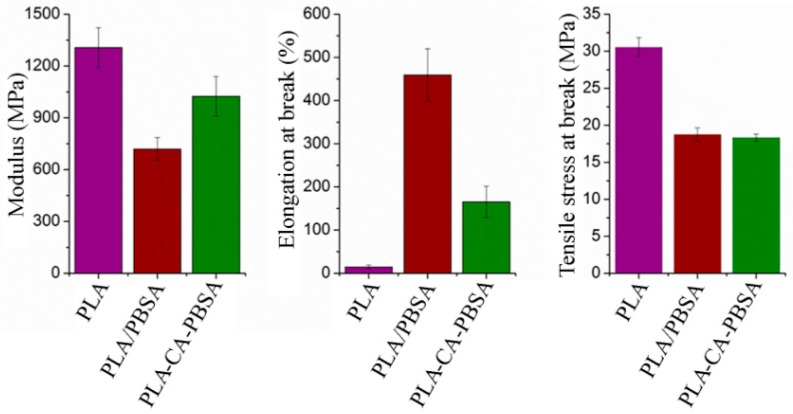
Mechanical properties of PLA, PLA/PBSA and PLA-CA-PBSA films.

Elongation at break was significantly increased from 14% to 460% after 20 wt % PBSA was blended with PLA (Figure 5). At the same time the modulus was decreased from 1300 to 720 MPa. The PBSA addition therefore reduced the modulus by half, while the elongation was increased more than 30 times. PLA-CA-PBSA showed only a slight decrease in modulus (1000 MPa), while the elongation still increased significantly (165%) as compared to neat PLA. The distinction between PLA/PBSA and PLA-CA-PBSA could be attributed to the different crystallization capacity and different glass transition temperatures. It can be concluded that the addition of the PBSA plasticizer increased elongation while it decreased the modulus, and stress at break of PLA, which is expected behavior after the addition of plasticizer.

**Figure 6:**
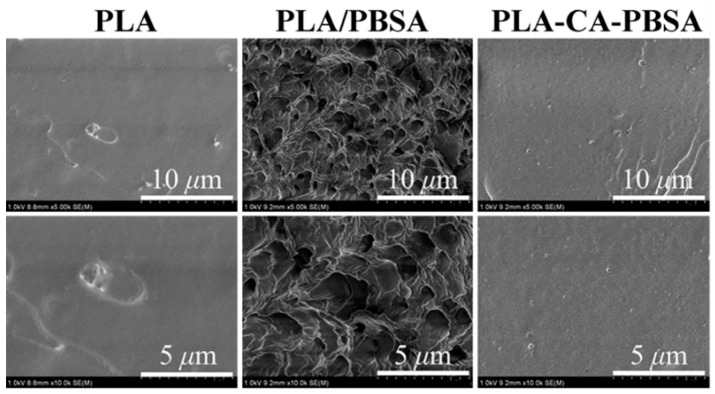
SEM micrographs of fractured surfaces of PLA, PLA/PBSA and PLA-CA-PBSA.

The morphology of fractured surfaces of PLA, PLA/PBSA and PLA-CA-PBSA films were investigated with SEM after they were fractured in liquid nitrogen, Figure 6. In agreement with previous studies, in which PLA was proven to be immiscible with high molecular weight PBS [22] and its copolymer PBSA [23,24]. Sea-island structures were observed on the PLA/PBSA fracture surfaces which is a typical phase separation morphology. Blending PBSA into PLA could still lower the *T*_g_, increase the chain mobility of PLA and change the materials tensile properties. These corroborate a partial miscibility between PBSA and PLA. However, neat PLA and PLA-CA-PBSA exhibited smooth surfaces. This phenomenon could probably be explained, with the combination of SEC and ^1^H NMR results, by two facts: coupling PBSA onto PLA could promote the dispersion of PBSA in PLA matrix and consequently lead to an improved miscibility, and coupled PBSA acted as a compatibilizer for un-coupled PBSA which increased PBSA miscibility in PLA matrix.

**Figure 7:**
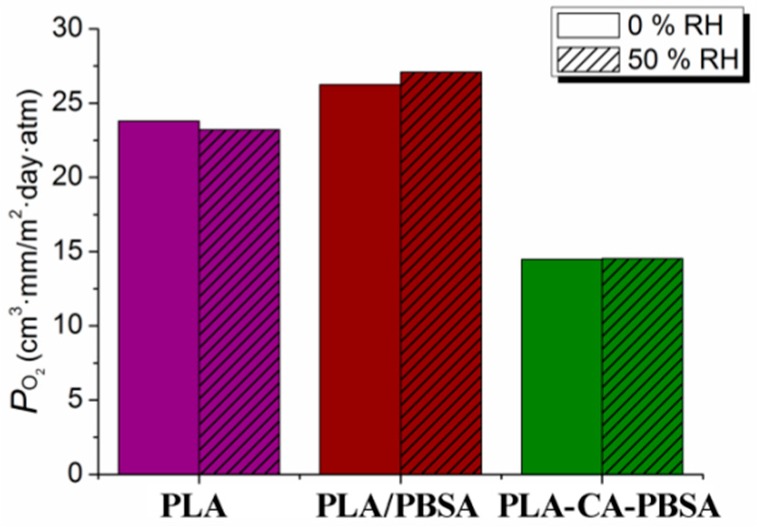
Oxygen permeability of PLA, PLA/PBSA and PLA-CA-PBSA films.

Gas permeability is an important property when bioplastics are used in packaging applications. The oxygen permeability slightly increased for PLA/PBSA probably due to the observed phase separation and lower *T*_g_, Figure 7. In contrast, decreased oxygen permeability was observed for PLA-CA-PBSA films. The relationship of crystallinity and barrier properties of PLA have been investigated in many studies, and it has been concluded that the gas/water permeability of semi-crystalline PLA cannot be simply explained by the variation in the degree of crystallinity [41,42,43,44,45]. The crystal polymorphism [42], the degree of space filling [44] and the rigid amorphous fraction [41,44] are all important parameters which influencing the barrier property of PLA. In the present study, the better oxygen barrier properties of PLA-CA-PBSA (*X*_c,PLA-CA-PBSA_ = 10.6%) compared to neat PLA (*X*_c,PLA_ = 2.06%) could be explained by the morphological features, as suggested in a previous study [44] in which PLA samples with the same degree of crystallinity but different degrees of space filling had different oxygen permeability. Similarly, as supported by the POM observations in Figure 3, PLA-CA-PBSA had strong nucleation ability leading to a large number of small crystals and a much higher degree of space filling as compared to PLA. This is combination with the higher degree of crystallinity could explain the reduced oxygen permeability observed for PLA-CA-PBSA. The commonly used polypropylene and polyethylene packaging have *P*_O_2__ of 50–100 and 50–200 cm^3^·mm/m^2^·day·atm at 23 °C under 50% or 10% RH, respectively [46], while PLA-CA-PBSA had *P*_O_2__ less than 15 cm^3^·mm/m^2^·day·atm. This is also significantly lower than the value 23 cm^3^·mm/m^2^·day·atm measured for pure PLA.

### 3.3. Hydrolytic Degradation and Migration Test of Compression Molded Film PLA, PLA/PBSA and PLA-CA-PBSA

**Figure 8:**
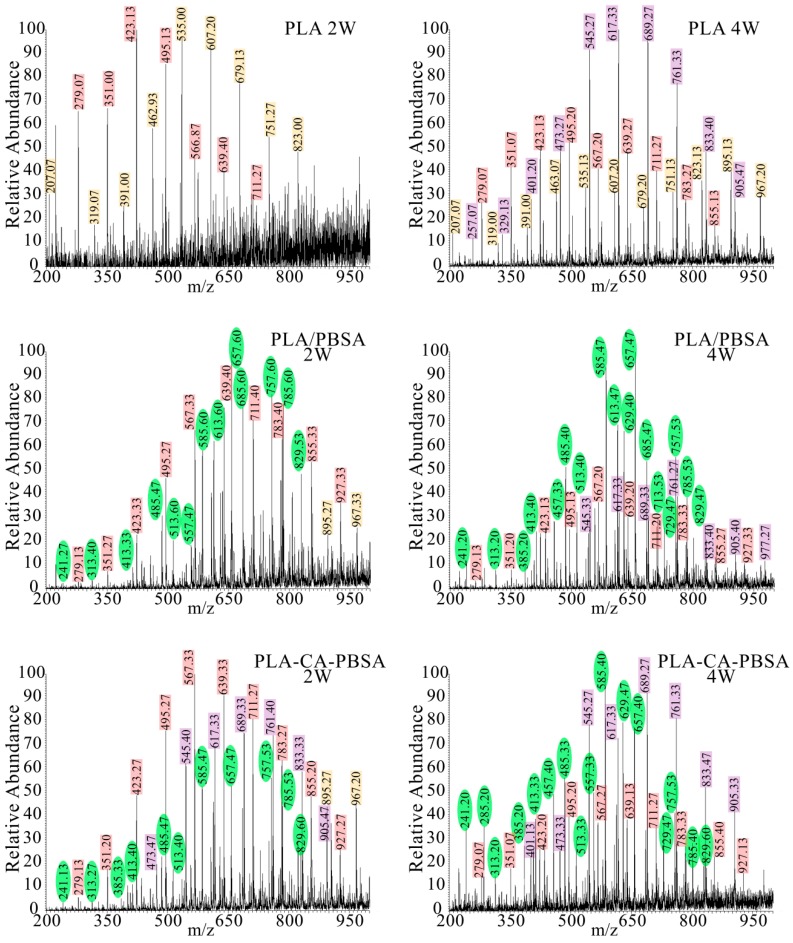
ESI-MS mass spectra after 2 and 4 weeks of hydrolysis at 60 °C (o-LA, yellow, pink and purple rectangle; PBSA, green oval).

The hydrolytic degradation and plasticizer migration behavior of the compression molded films were studied for up to 4 weeks in water at 60 °C. After 2 and 4 weeks of hydrolysis, linear lactic acid oligomers were detected in all samples. The main series of peaks in the mass spectra of neat PLA after 2 weeks of hydrolysis were adducts of linear lactic acid oligomers [o-LA + 2Na]^+^ marked with pink rectangles and [o-LA + 3Na + H_2_O]^+^ marked with yellow rectangles, Figure 8. After 4 weeks of hydrolysis, an additional series of adducts [o-LA + Na]^+^ was detected and the adducts were marked with purple rectangles. In contrast to neat PLA, after 2 weeks of hydrolysis, the main series of peaks in the case of PLA/PBSA were both [o-LA + 2Na]^+^ and PBSA or PBSA hydrolysis products which were marked with green ovals. This indicates that PBSA migrated from the PLA/PBSA blends already after 2 weeks of hydrolysis and this migration increased further after 4 weeks. The main series of peaks after 4 weeks of hydrolysis were adducts of PBSA. PLA-CA-PBSA had a different hydrolysis product pattern compared to PLA/PBSA. Adducts of linear lactic acid oligomers were the main series of peaks in the mass spectrum after 2 weeks. Unreacted PBSA or PBSA hydrolysis products were detected but with low intensity. After 4 weeks, adducts of linear lactic acid oligomers were still the main series of peaks. The intensity of the PBSA adducts increased as hydrolysis proceeded, especially in the low molecular weight range. This is perceived as evidence that the increasing intensity of PBSA was the consequence of PBSA hydrolysis instead of the migration of unreacted PBSA.

## 4. Conclusions

Plasticized PLA was obtained by blending PLA with low molecular weight PBSA and coupling PBSA to carboxylic-functionalized PLA. As expected, blended PBSA and grafted PBSA both increased the flexibility and decreased the *T*_g_ of PLA significantly. Elongation at break was remarkably increased from 14% to 460% when PBSA was blended with PLA. PLA-CA-PBSA had 165% as its elongation at break, which was 10 times higher than neat PLA. Together with the immiscible molten droplets of PBSA or PBSA coupling points which acted as nucleating agents, both PLA/PBSA and PLA-CA-PBSA showed higher crystallinity in DSC measurement. An increased nucleation ability and crystal growth rate of plasticized PLA conformed with POM observation of isothermally crystallized samples. PLA-CA-PBSA showed the highest nucleation ability, followed by PLA/PBSA. WAXD results for isothermally crystallized materials indicated that the observed crystals originated from crystallization of PLA chains. Moreover, enhanced gas barrier properties were achieved after PLA was coupled with PBSA, as a result of higher crystallinity and enhanced miscibility between PLA and PBSA. Last but not least, higher migration resistance of PLA-CA-PBSA in comparison with PLA/PBSA was confirmed by hydrolytic degradation study.

## Figures and Tables

**Table 1:** Number average molecular weight (*M*_n_), weight average molecular weight (*M*_w_), z-average molecular weight (*M*_z_) and polydispersity index (*Ð*) of the different PLA films.

Sample	*M*_n_ (g/mol)	*M*_w_ (g/mol)	*M*_z_ (g/mol)	*Ð*
**PLA**	160,000	279,000	411,000	1.7
**PLA-CA**	150,000	257,000	379,000	1.7
**PLA/PBSA**	138,000/1900	254,000/6700	388,000/13,300	1.8/3.4
**PLA-CA-PBSA**	181,000/11,900	279,000/17,200	387,000/23,200	1.5/1.4

**Table 2:** Thermal properties of PLA, PLA/PBSA and PLA-CA-PBSA.

**Sample**	*T*_cc_ * (°C)	*T*_m_ * (°C)		*Χ*_c_ * (%)	*T*_mc_ ** (°C)
**PLA**	-	142.9 ± 0.2		2.1 ± 0.2	-
**PLA/PBSA**	79.3 ± 0.8	133.0 ± 1.2	145.2 ± 0.2	8.4 ± 0.3	83.1 ± 1.2
**PLA-CA-PBSA**	76.3 ± 0.2	131.1 ± 0.2	145.1 ± 0.0	10.6 ± 1.0	80.4 ± 0.3
**Sample**	*T*_g_ *** (°C)	*T*_cc_ *** (°C)		*T*_m_ *** (°C)	
**PLA**	44.3 ± 0.7	-		142.2 ± 0.1	
**PLA/PBSA**	18.3 ± 0.5	74.1 ± 1.8		144.1 ± 0.2	
**PLA-CA-PBSA**	24.4 ± 0.5	84.9 ± 0.2		127.0 ± 0.2	141.6 ± 0.2

* *T*_cc_, *T*_m_ and *Χ*_c_ were collected from the first heating scan; ** *T*_mc_ represents the melt crystallization temperature during cooling; *** *T*_g_, *T*_m_ and *T*_cc_ were determined from the second heating scan.

## Abbreviations

The following abbreviations are used in this manuscript:

PLA	polylactide
PBSA	poly(butylene succinate-co-adipate)
CA	crotonic acid
PEG	poly(ethylene glycol)
Acrylated PEG	acrylated poly(ethylene glycol)
PHB	poly(3-hydroxybutyrate)
PBA	poly(butylene adipate)
PBS	poly(butylene succinate)
TPP	triphenyl phosphite
TIP	titanium isopropoxide
PLA-CA	crotonic acid grafted PLA
PLA/PBSA	PLA/PBSA blends
PLA-CA-PBSA	PBSA coupled PLA-CA
